# Gut microbiome: New biomarkers in early screening of colorectal cancer

**DOI:** 10.1002/jcla.24359

**Published:** 2022-03-21

**Authors:** Peng Zhou, Dongxue Yang, Desen Sun, Yuping Zhou

**Affiliations:** ^1^ Department of Biochemistry and Molecular Biology, and Zhejiang Key Laboratory of Pathophysiology School of Medicine Ningbo University Ningbo China; ^2^ Department of Gastroenterology The Affiliated Hospital of Medical School Ningbo University Ningbo China; ^3^ Institute of Digestive Disease of Ningbo University Ningbo China

**Keywords:** biomarker, colorectal cancer, diagnosis, gut microbiome, progress

## Abstract

**Background:**

Certain “star intestinal bacteria” have been found to act as a contributor to the development of colorectal cancer (CRC). Besides, given that the gut microbiome can be detected in a diverse range of samples (stool, tissue, blood, etc), it is categorized into fecal microbiome, blood microbiome, and tissue microbiome.

**Methods:**

To provide an overview of the recent research progress, this review summarizes the characteristics of the gut microbiome in different samples at each stage of CRC and their screening efficiency.

**Results:**

The screening models constructed from different sample microbiomes (healthy/colorectal adenoma, healthy/CRC, and colorectal adenoma/CRC) have both strengths and constraints in terms of biomarker reproducibility and area under the receiver‐operating characteristic curve (AUC) of the screening models. Many bacteria, such as Bifidobacteria, Fusobacterium nucleatum (F. n), Geotrichum candidum, Porphyromonas asaccharolytica, Escherichia coli, Rhodococcus, Anaerostipes caccae, Enhydrobacter, Lachnoclostridiumsp. m3, Bacteroides clarus, Clostridium hathewayi, Ruminococcaceae, Bacteroides thetaiotaomicron, Culinariside, and enterotoxigenic Bacteroides fragilis (ETBF), show favorable diagnostic efficacy in early screening of colorectal cancer.

**Conclusions:**

This review highlights stool, blood, tissue, and bowel fluid are the main sample sources for biomarkers, each with its own advantages and limitations. Moreover, other samples such as extracellular vesicles and biofilms also should been deserved further attention.

## Graphical abstract

The gut microbiome can be categorized into fecal microbiome, blood microbiome, and tissue microbiome. This review summarizes the characteristics of the gut microbiome in different samples at each stage of CRC and their screening efficiency to provide an overview of the recent research progress.

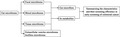

## 1 INTRODUCTION

Colorectal cancer (CRC) includes carcinogenesis of the colon and rectum, with high morbidity and mortality. According to the latest epidemiological data in 2020, there have been a total of 1,880,700 new cases of CRC and 915,800 deaths in the world, ranking third and second among all malignancies.^1^ In China, according to the data published in 2020, the morbidity and mortality rate ranks third and fifth, respectively, among malignant tumors.^2^ In addition, the patient's prognosis is closely related to the stage of CRC development, with a 5‐year survival rate ranging from approximately 80% in stage I patients to just over 10% in stage IV patients.^3^ Thus, it is urgent to identify risk factors/biomarkers for CRC early screening.

Existing clinical screening tools for CRC include gastrointestinal endoscopy, fecal occult blood test, fecal immunochemical test, tumor markers, and abdominal computed tomography scan. However, the above screening approaches have problems with patient compliance, as well as sensitivity and specificity of the tests.^4^ Several factors contribute to the development of cancer, such as diet, host genetics, gender, and age, and the gut microbiome is attracting increasing attention from researchers. For example, some "star bacteria" play an important role in the development of CRC, such as *Fusobacterium nucleatum*, *Escherichia coli*, and the *enterotoxigenic Bacteroides fragilis*.^5^ Thus, gut microbiome may be used as a promising biomarker for early screening of CRC.^6^

**FIGURE 1:**
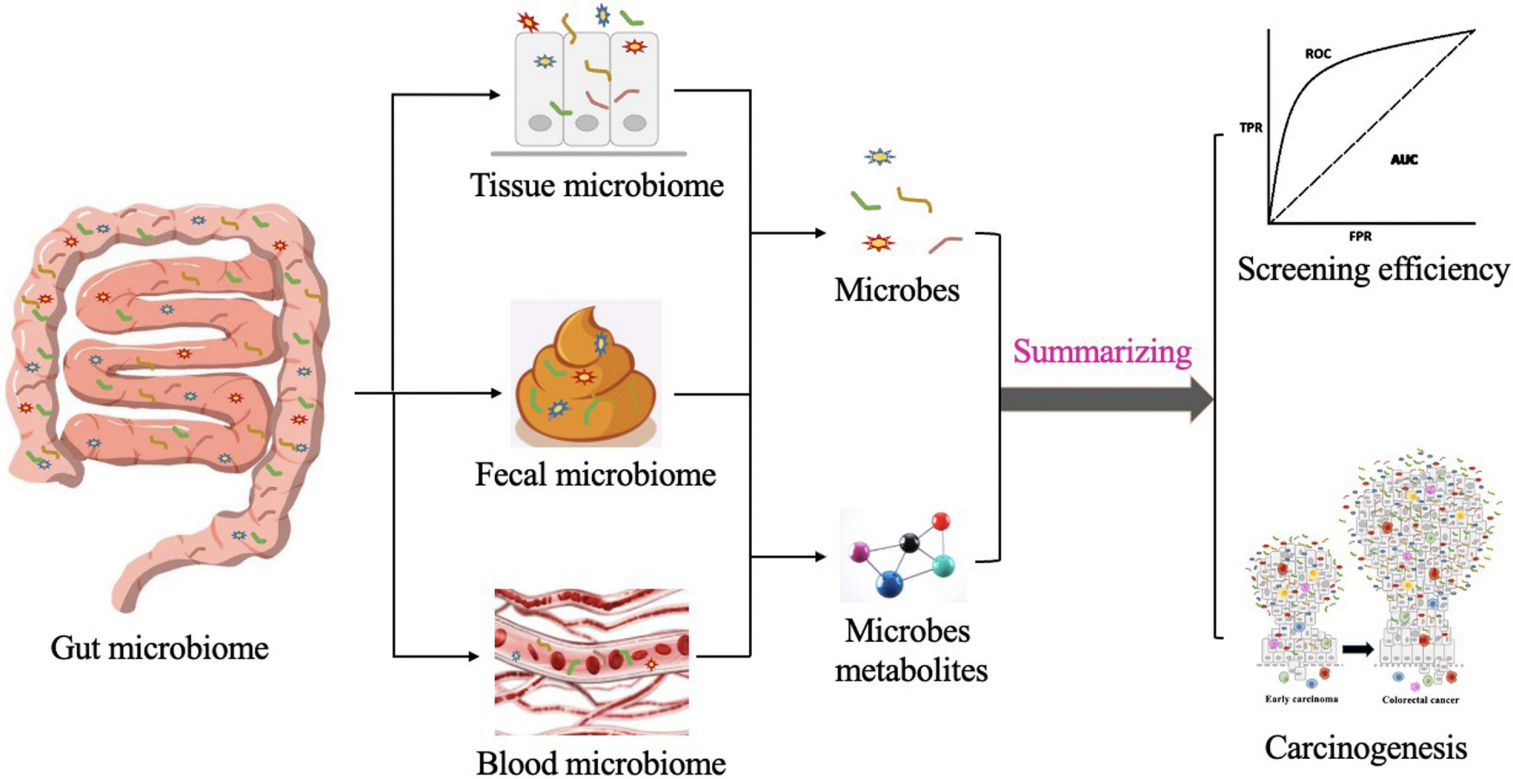
Gut microbiome: New biomarkers in early screening of colorectal cancer

There are horizontal and vertical translocation of gut microbiome, the horizontal translocation is the transfer of the gut microbiome among different locations in the digestive tract; the vertical translocation is the transfer of the gut microbiome from the intestinal lumen to the deep mucosa, as well as the transfer of the entire host through the blood circulation.^7^ With the advancement of the microbiomic research, scientists have gradually shifted their preference on choosing samples from feces to other samples such as blood and tissues. At present many studies are available in China and abroad showing the use of different samples from different sources such as feces, blood, tissues, and intestinal fluids to detect intestinal microflora and their metabolites, to construct stage‐specific prediction models for CRC and achieve an early screening. This review summarizes and compares recent advances in early CRC screening with different types of samples (Figure 1).

## 2 FECAL MICROBIOME AND EARLY DETECTION OF CRC

Since fecal samples are non‐invasive and convenient to obtain, the study of fecal microbiome in early CRC screening has been conducted earlier and relatively more studies have been reported. In most cases, the development of CRC follows the following progression of events: "normal intestinal mucosal epithelium—hyperplastic polyps (non‐progressive adenomatous polyps)—progressive adenomatous polyps—early carcinoma—colorectal cancer (stages I, II, III, and IV)."^8^ Pathogenic intestinal bacteria such as *Fusobacterium nucleatum*, *Escherichia coli*, and *enterotoxigenic Bacteroides fragilis* play an important role in this progression.^5^ For example, the production of the adhesin FadA by *Fusobacterium nucleatum* is involved in the activation of the β‐linked protein‐Wnt pathway and facilitates the development of CRC.^9^, ^10^ A number of studies confirmed the significant difference in the fecal microbiome of patients at different CRC stages.^11^, ^12^, ^13^, ^14^ The variety of pathogenic intestinal bacterial species and number increase continuously during CRC development, for example, *Fusobacterium nucleatum* is significantly abundant in the feces of healthy individuals, colorectal adenoma (CRA) patients, and CRC patients.^15^, ^16^ Yachida S et al divided 616 participants who underwent colonoscopy into a healthy control group (*n* = 251), a multiple polypoid adenoma group (MP, *n* = 67), intra‐mucosal carcinoma group (S0, *n* = 73), CRC stage I/II group (SI/II, *n* = 111), and CRC stage III/IV group (SIII/IV, *n* = 74) and found that the abundance of *Fusobacterium nucleatum* in feces increases significantly from the S0 to the SIV stage.^11^ In addition, the abundance of *Vibrio vulnificus* and *Actinobacterium lysogenicum* and their associated metabolites deoxycholic acid, branched‐chain amino acids and phenylalanine also increased significantly in the feces of the MP and S0 groups. This study demonstrates the heterogeneity of the fecal microbiome in the different stages of CRC and numerous studies reached similar consistent conclusions.^11^, ^12^, ^13^, ^14^ The apparent heterogeneity of the fecal microbiome in different CRC stages provides an experimental basis for its use as an early biomarker in the detection of CRC.

Further studies found that the fecal microbiome has the potential to predict the presence of mutations in CRC‐related genes, suggesting important implications in the identification of high‐risk groups and even for the selection of specific treatment regimens. The evolution of CRC is influenced by environmental and genetic factors, and among the latter, 12–35% of CRC patients have a genetic background.^17^ Indeed, the first‐degree relatives of CRC patients are at a higher risk (2~4‐fold) of developing CRC than the general population, and if two or more close relatives (parents or siblings) in the family have CRC, the other members of the family are at high risk of CRC; thus, genetic factors determine the risk of CRC to some extent. Familial adenomatous polyposis (FAP) is an autosomal dominant disorder with a high cancer rate, and mutations in the adenomatous polyposis coli (APC) gene are one of its pathogenic mechanisms.^18^ A study on 35 patients with FAP revealed the presence of a specific microbiome in the feces of these patients with APC mutations.^19^ In addition, two studies indicated that the fecal microbiome has the potential to predict the presence of mutations in BRAF and KRAS genes, the latter being closely associated with targeted therapies.^20^, ^21^ The above studies suggest that the fecal microbiome may potentially identify high‐risk groups carrying mutations in CRC‐related genes, providing a reference for the selection of the optimal treatment for CRC patients.

The heterogeneity of the fecal microbiome in different CRC stages represents the basis for exploring biomarkers for the early detection of CRC. Some investigators developed diagnostic models using different microbiome markers in feces or in combination with other clinical screening tools such as fecal immunochemical tests and fecal miRNA quantification and found substantial predictive factors for CRA stage.^15^, ^22^, ^23^ However, since the composition of the intestinal microflora is influenced by various factors such as dietary habits, geography, obesity and gender, and evident differences are found in the predictive markers derived from different studies performed in different places, raising the issue of reproducibility of the predictive markers. A recent multicenter study confirmed the reproducibility of the predictive markers by collecting fecal macrogenomic sequencing data from 775 samples and constructing stage‐specific predictive models for healthy/CRA (AUC = 0.80) and CRA/CRC (AUC = 0.89) based on 11 and 26 bacterial predictive markers, respectively, and in another 2 independent cohorts they confirmed the AUC with values of 0.78 and 0.84.^24^ In addition, several studies identify reproducible predictive markers by meta‐analysis of different ethnic/regional datasets, and the constructed diagnostic models for healthy/CRA, and CRA/CRC classification have high sensitivity and specificity (Table 1),^16^, ^24^, ^25^, ^26^, ^27^, ^28^ suggesting that the fecal microbiome has a promising clinical potential to detect CRC in its early stage. Among various screening models, the ones constructed by Wu Y et al only used bacteria as markers, which is simpler and more convenient to apply in clinical practice,^24^ with the advantage of promotion and the acceleration of the clinical translation of the fecal microbiome in the early detection of CRC.

**TABLE 1:** Studies related to intestinal microflora and its metabolites in the early detection of colorectal cancer

Literature	Research type	Population	Samples	Biomarkers	Screening models (AUC, sensitivity, specificity)
Wang H F, et al (2016)^38^	Single‐center clinical study	Total 608: 258 CRC 150 CRA 200 Normal	Blood	anti‐F.n IgA, anti‐F.n Ig G	**anti‐F.n IgA+CEA:** Normal/CRC (AUC = 0.848, sensitivity 53.1%, specificity 96.4%) **anti‐F.n IgA+CEA+CA19‐9:** Normal/CRC (AUC = 0.743, sensitivity 40.0%, specificity 94.2%)
Tarallo S, et al (2019)^23^	Single‐center clinical study	Total 80: 29 CRC 27 CRA 24 Normal	Stool	Bacterial DNA, Bacterial sRNA (*Escherichia coli*, Bifidobacteria, etc.) and human Hsa‐miRNA	**Bacterial DNA+Bacterial sRNA:** Normal/CRA (AUC = 0.42), CRA/CRC (AUC = 0.71), Normal/CRC (AUC = 0.83) **Bacterial DNA+Bacterial sRNA+Human Hsa‐miRNA:** Normal/CRA (AUC = 0.47), CRA/CRC (AUC = 0.74), Normal/CRC (AUC = 0.87)
Thomas A M, et al (2019)^26^	Meta‐analysis	Total 764: 313 CRC 143 CRA 308 Normal	Stool	16 CRC‐associated pathogenic bacteria (Fusobacterium nucleatum, Geotrichum candidum, Porphyromonas asaccharolytica, etc.)	**16 CRC‐associated pathogenic bacteria:** **Validation queue 1:** Normal/CRC (AUC = 0.91) **Validation queue 2:** Normal/CRC (AUC = 0.77) Normal/CRA (Max AUC = 0.58)
Zhang X, et al (2019)^16^	Systematic review and Meta‐analysis	Total 10 researchs Total 4354: 1450 CRC 656 CRA 2248 Normal	Stool	Fusobacterium nucleatum (F.n)	**F.n:** Normal/CRC (AUC = 0.80, sensitivity 71%, specificity 76%) Normal/CRA (AUC = 0.60, sensitivity 36%, specificity 73%)
Huang Y. (2019)^37^	Single‐center clinical study	Total 480: 240 CRC 160 CRA 80 Normal	Blood, stool	Fusobacterium nucleatum (F.n), enterotoxigenic Bacteroides fragilis (ETBF)	**Stool F.n:** Normal/CRC (AUC = 0.689) **Stool ETBF:** Normal/CRC (AUC = 0.593) **Stool F.n+Stool ETBF:** Normal/CRC (AUC = 0.731) **Stool F.n+Stool ETBF+Blood F.n:** Normal/CRC (AUC = 0.763)
Gao R, et al (2020)^25^	Multi‐center clinical study	Total 792: 155 CRC 195 CRA 442 Normal	Stool	18 CRC‐associated genera (Escherichia coli, Rhodococcus, Anaerostipes caccae, etc.)	**18 CRC‐associated genera:** Normal/CRA (AUC = 0.616), CRA/CRC (AUC = 0.725), Normal/CRC (AUC = 0.858) **18 CRC‐associated genera+FIT:** Normal/CRA (AUC = 0.717), CRA/CRC (AUC = 0.781), Normal/CRC (AUC = 0.992)
Mo Z, et al (2020)^28^	Meta‐analysis	Total 15 researchs Total 2099: 1107 CRC 492 CRA 500 Normal	Stool, tissue	61 CRC‐associated genera (Enhydrobacter, Fusobacterium nucleatum, etc.)	**61 CRC‐associated genera:** **Stool:** Normal/CRC (AUC = 0.8), Normal/CRA (AUC = 0.69) **Carcinoma tissue:** Normal/CRC (AUC = 0.96), Normal/CRA (AUC = 0.91) **Adjacent normal tissue:** Normal/CRC (AUC = 0.95), Normal/CRA (AUC = 0.89)
LiangJ Q, et al (2021)^15^	Multi‐center clinical study	Total 676: 210 CRC 201 CRA 265 Normal	Stool	Fusobacterium nucleatum (F.n), Lachnoclostridiumsp.m3 (m3), Bacteroides clarus, Clostridium hathewayi	**F.n:** Normal/CRC (AUC = 0.856), Normal/CRA (AUC = 0.619) **m3:** Normal/CRC (AUC = 0.767), Normal/CRA (AUC = 0.669) **F.n+m3+Bacteroides clarus+Clostridium hathewayi:** Normal/CRC (AUC = 0.884, sensitivity 84.9%, specificity 83.3%) Normal/CRA (AUC = 0.669, sensitivity 38.6%, specificity 98.6%)
Wu Y, et al (2021)^24^	Multi‐center clinical study	Total 1056: 217 CRC 306 CRA 252 Normal	Stool	8 CRA‐associated genera and 24 CRC‐associated genera (Ruminococcaceae, Bacteroides thetaiotaomicron, etc.)	**8 CRA‐associated genera+age+Gender+BMI:** Normal/CRA (AUC = 0.80, sensitivity 82.0%, specificity 62.0%) **24 CRC‐associated genera+age+gender+BMI:** Normal/CRC (AUC = 0.89, sensitivity 66.0%, specificity 90.0%)
Chen F, et al (2021)^33^	Multi‐center clinical study	Total 484: 202 CRC 46 CRA 167 Normal (69 Not recorded)	Blood	8 gut microbiome‐associated metabolites (Culinariside, 14‐HDoHE, etc.)	**8 gut microbiome‐associated metabolites:** Normal/CRA (AUC = 0.84, sensitivity 63.2%, specificity 84.9%) Normal/CRC(I/II) (AUC = 0.93, sensitivity 88.2%, specificity 84.9%) Normal/CRC(III/IV) (AUC = 0.91, sensitivity 84.2%, specificity 84.9%)

Abbreviations: AUC, area under curve; CA19‐9, carbohydrate antigen 19‐9; CEA, carcinoembryonic antigen; CRA, colorectal adenoma; CRC, colorectal cancer; ETBF, enterotoxigenic B. fragilis; F.n, fusobacterium nucleatum.

## 3 BLOOD MICROBIOME AND EARLY DETECTION OF CRC

Current studies confirmed the involvement of the intestinal pathogenic bacteria in the downregulation of the expression of proteins involved in the intestinal epithelial tight junction and the disruption of the function of the intestinal epithelial barrier, leading to the translocation of pathogenic bacteria and their metabolites in the intestinal lumen into the blood stream.^29^ Experiments in mice revealed that intestinal bacteria deliver biologically active molecules to various organs of the body (including intestine–liver axis and intestine–brain axis) by releasing vesicles into the blood stream.^30^ The above findings suggest the presence of an intestinal‐derived microbiome in the blood, providing a theoretical basis in the use of blood as a sample for the early diagnosis of CRC.

A recent study published in the journal nature analyzed blood and tissue samples from patients with 33 types of cancer (10,000 cases and 18,000 samples) and found the presence of unique gut‐derived pathogenic bacterial DNA in the blood that could be used to distinguish between different cancer types,^31^ and the authors realized that this potential microbiome‐based tumor diagnostic tool deserves further exploration. As for CRC, which is a common gastrointestinal malignancy, the exploration of pathogenic bacterial DNA and metabolites in the blood associated to CRC is also one of the current hotspots of research.^32^ Chen F et al performed macrogenomic and metabolomic analyses of serum from healthy individuals, CRA, and CRC patients and found a total of 885 differential metabolites associated with intestinal bacterial in the serum, eventually identifying eight serum metabolites with reproducibility, thus used to construct healthy/CRA (AUC = 0.84) and healthy/CRC (AUC = 0.93) categorical diagnostic models.^33^

Some common intestinal bacterial metabolites in the blood, such as short‐chain fatty acids, bile acids, and oxotrimethylamine, have a potential role as biomarkers for the early detection of CRC.^34^, ^35^, ^36^ A study by Huang Y et al revealed the role of *Fusobacterium nucleatum* in mediating colorectal carcinogenesis through the histidine metabolic pathway, leading to increased concentrations of tumor‐associated metabolites such as 12a hydroxy3oxycholic acid and phosphorylcholine in the blood.^37^ In addition, they combined *Fusobacterium nucleatum* in the blood with *Fusobacterium nucleatum* and fecal *enterotoxigenic Bacteroides fragilis* and constructed a predictive model with a favorable CRC detection ability (AUC = 0.763). Furthermore, pathogenic bacteria can induce an immune response and produce antibodies. Wang H F et al found that the IgA and IgG concentrations against *Fusobacterium nucleatum* in the serum of CRC patients are significantly higher than those in the CRA and healthy population, and the AUC for antibodies combined with the carcinoembryonic antigen and glycoantigen 19–9 was 0.848 and 0.743.^38^

The above studies suggest that the blood microbiome has good predictive ability to detect CRC in its early stage. However, most of the studies on blood microbiome in the early detection of CRC are performed on a limited number of samples compared with those performed on fecal microbiome, and larger prospective multicenter cohort studies are needed to find more reliable biomarkers for an early detection of CRC.

## 4 INTESTINAL MUCOSAL TISSUE MICROBIOME AND EARLY DETECTION OF CRC

The current routes to access the bowel tissue are surgical and gastrointestinal endoscopy, and both are invasive procedures, thus limiting the clinical use of tissues as samples for the early detection of CRC. However, tissue microbiome still has an important research value since it is more relevant to the pathological processes involved in the development of CRC. Consistent with the findings of fecal and blood microbiomes in the early detection of CRC, the heterogeneity of the tissue microbiome is present in different CRC stages as well as in the microbiomes of tumor tissues from different intestinal locations, which is important to support their potential role in the early detection of CRC.

Yamamoto S et al found *Fusobacterium nucleatum* in the surface and deep tumor tissues of 45.7% and 32.6% of CRC patients, respectively, and its abundance increases with the development of CRC stage (from 5.9% in CRA to 81.8% in CRC stage III/IV).^21^ Moreover, the heterogeneity of the tissue microbiome in different CRC stages indirectly suggests that the fecal microbiome and blood microbiome of CRC stage heterogeneity. One study analyzed the microbial diversity in the tumor tissues from different intestinal locations and found that the proximal intestinal cancer tissues are significantly richer in intestinal bacteria than the distal intestinal cancer tissues.^39^ In 2012, Tjalsma H et al were the first to propose a “driver‐passenger” model to explain the “adenoma‐carcinoma” progression of CRC.^40^ The model suggests that driver bacteria promote the colonization of passenger bacteria by altering the intestinal microenvironment, and differences in the types of driver and passenger bacteria are found at different stages of CRC development. Since then, several studies based on the "driver‐passenger" model have reached consistent conclusions.^9^, ^41^, ^42^ Wang Y et al sequenced CRC cancer and paraneoplastic tissues and identified 4 genera and 2 families of potential driver bacteria and 14 genera and 14 families of potential passenger bacteria, suggesting their potential role as predictive markers for the early diagnosis of CRC.^9^

## 5 INTESTINAL FLUID MICROBIOME AND EARLY DETECTION OF CRC

The intestinal fluid, known as intestinal lavage fluid, is also a sample source to detect intestinal bacteria. Shen W et al compared the microbiome of the intestinal fluid with that of feces in healthy controls and CRC patients and found that the pathogenic bacteria in the intestinal fluid are more abundant than in feces.^43^ In China, Zhang B et al analyzed the structure of the intestinal fluid bacteriome of CRA and healthy individuals and found a heterogeneity in the intestinal fluid microbiome among different CRC stages.^44^ They found that the abundance of *Bacillus spp*., *Pseudomonas spp*., and *Aspergillus spp*. in the intestinal fluid of CRA patients is significantly higher than that in healthy individuals. In addition, a study using both the intestinal fluids and tissue samples found the presence of pathogenic bacteria in the intestinal fluid, which are closely associated with the development of CRC and do not differ significantly from those in tissue samples.^45^ The above studies confirm that the use of intestinal fluid as a sample for the detection of intestinal bacteria is a feasible choice.

Not many current studies are available on the microbiome of bowel fluid for the early detection of CRC, mainly due to the limited methods for the collection of bowel fluids. At present, endoscopic aspiration is the main approach to obtain the intestinal fluid, and although its risk is significantly reduced compared with the endoscopic biopsy, it is still an invasive procedure. Some scientists developed non‐invasive sampling devices for the collection of bowel contents,^46^, ^47^, ^48^ such as Cui J et al who invented an orally swallowable capsule that can be positioned in the gastrointestinal tract to collect bowel fluid and deliver drugs.^48^ The feasibility of the intestinal fluid collection may be the basis to collect samples for the early detection of CRC. In the future, relevant studies will be gradually carried out, as more non‐invasive sampling devices are developed and disseminated. Thus, the bowel fluid has the potential to become a new bioassay sample for the early detection of CRC.

## 6 CONCLUSION

Research on the relevance of intestinal bacteria and their metabolites in CRC has been a hot topic in recent years, and significant progress has been made in their use as markers for the early detection of CRC. Stool, blood, tissue, and bowel fluid are the main sample sources for biomarkers, each with its own advantages and limitations. The fecal microbiome has been more intensively studied for the early detection of CRC and has the advantages of high specificity, sensitivity, and reproducibility, with the potential to screen people at high risk of CRC‐related mutations. Blood is a commonly used clinical test sample. The blood microbiome has good promotion advantages, and the predictive models constructed have high sensitivity and specificity, but the reproducibility of markers needs to be further determined by multicenter studies with larger samples. As a novel source of samples and in direct contact with the intestinal epithelium, enteric fluid showed no significant difference in pathogenic bacteria compared to tissue and feces, but the limitations of the techniques for clinical collection and the cumbersome process of extracting enteric fluid via endoscopy as well as the easy contamination of the sample make difficult to conduct relevant studies at present. Therefore, there is a need to develop more non‐invasive devices to allow the collection and study of bowel fluid in the future. Moreover, other samples such as extracellular vesicles and biofilms have also been initially studied and explored in the early detection of CRC and deserve further attention.^49^, ^50^ In the future, more animal‐level and cellular‐level mechanistic studies should be performed to provide an experimental basis and theoretical foundation for the collection of various samples to use in studies on the early detection of CRC. Finally, the potential use of microorganisms such as fungi and viruses in the gut as biomarkers for the early detection of CRC is also a topic that needs to be evaluated in the future.

## CONFLICT OF INTEREST

The authors declare no conflict of interest.

## Data Availability

All data included in this study are available upon request by contact with the corresponding author.
